# Dural Closure Training With Prototyped Model

**DOI:** 10.7759/cureus.61688

**Published:** 2024-06-04

**Authors:** Hanin El Husseini, Joseph F Chenisz da Silva, André Giacomelli Leal, Lorena Maria Dering, Ricardo Ramina, Igor Alves da Silva, Mohamed El Husseini

**Affiliations:** 1 Medicine, Positivo University, Curitiba, BRA; 2 Neurosurgery, Neurological Institute of Curitiba (Hospital INC), Curitiba, BRA; 3 Technology and 3D Printing, Neurological Institute of Curitiba (Hospital INC), Curitiba, BRA

**Keywords:** 3d printing, neurosurgery, durotomy repair, simulation mode, resident education

## Abstract

Introduction: The hermetic closure of the dura mater is a critical step in neurosurgical training, often undervalued but crucial to preventing serious complications such as cerebrospinal fluid (CSF) leaks leading to meningitis and death. Inadequate closure, often due to insufficient training, can result in challenging complications, including prolonged hospitalization and reoperation.

Objective: To address the deficiencies in dural closure training, this study aims to describe a 3D prototype for simulating post-craniotomy dura mater suturing. The objective is to reduce the incidence of CSF leaks and improve the training of neurosurgery residents.

Design: The study involves the creation of a 3D prototype based on magnetic resonance imaging and computed tomography scans. The additive manufacturing of structures is performed using ABS filament, and a silicone rubber membrane is used to simulate the meningeal dura mater. Neurosurgery residents undergo training using this model, and the effectiveness is evaluated.

Setting: The study is conducted at the Institute of Neurology of Curitiba (Hospital INC), focusing on neurosurgery residents from the first to fifth year of residency.

Participants: Seven residents participate in the study, with varying levels of experience in dural closure procedures. The training involves a simulated surgical environment using the 3D prototype.

Results: After training, residents show improvements in confidence and theoretical knowledge related to dural closure. Binary questions indicate a strong desire for more practical training on dural closure, with 85.7% believing in the essential role of 3D molds in their neurosurgery training.

Conclusion: The study highlights the importance of adequate training for dural closure to prevent serious complications in neurosurgery. The use of 3D simulation models, despite some limitations, proves to be an effective educational strategy. The emerging technology of bioprinting holds promise for further enhancing simulation materials, bringing medical education closer to realistic tissue replication.

## Introduction

The hermetic closure of the dura mater is an essential step in the training of any neurosurgeon; it is an extremely important moment, often undervalued by neurosurgeons in training. The consequence of inadequate closure, often due to insufficient training, can lead to serious complications, such as cerebrospinal fluid (CSF) leak with consequent meningitis and even death [1,2]. Among the complications in neurosurgery, CSF leak is one of the most challenging, often leading to prolonged hospitalization and reoperation. Furthermore, a fistula can lead to other complications such as an increased possibility of infection [3].

In the modern days, the procedure is performed by direct suture, which is most commonly used, or indirect suture, which involves the use of biomaterials (grafts, protein-based adhesives, bacterial cellulose membrane), non-biological (synthetic) materials and composite materials (electrospinned membranes, dermal fibroblasts and mussel adhesive proteins). In a study that reviewed 3822 cases of dural repair, it was concluded that the total failure rate of direct suture repair was lower than that of indirect repair [4], reinforced by a systematic review of 11 studies in which the incidence of complications in cases of inadequate closure, such as cerebrospinal fluid fistula, was the lowest in primary suture [5]. Nevertheless, despite being the initial method of choice, direct suturing has a high failure rate of 5% to 9%, being, in most cases, a consequence of the surgeon's inexperience [6].

In this sense, since the main cause of defects in the dura mater is incidental [7], it is essential that doctors undergoing medical residency training have adequate training to avoid complications. These complicated scenarios, when neglected, can evolve into relatively simple adverse situations such as adult migraine, nausea, photophobia; and even serious secondary diseases such as intracranial hemorrhage, pseudocyst formation, cerebrospinal fluid leaks and serious infectious, that are often difficult to treat and correct [8,9]. Moreover, in a meta-analysis of 23 studies [10], the incidence of dural injury related to neurosurgery was 5.8%, reinforcing the need for adequate training of future surgeons.

Notwithstanding, there is concern that, within the current scenario, residents do not receive the necessary preparation, mainly due to recent advents of reduced working hours, limitation of residents' peri-operative work, especially in an adequate laboratory, in addition to the difficulty of anatomical pieces due to bureaucracy specific to each region [11-13].

Consequently, as an alternative, surgical simulation practices witnessed rapid expansion [13]. To address the deficiencies described, the Institute of Neurology of Curitiba (Hospital INC) worked to create a dural closure model. This involves training in post-craniotomy dura mater suturing, aiming to reduce the incidence of cerebrospinal fluid leaks and consequently improve the training of doctors in specialization.

Accordingly, the present article aims to describe a 3D prototype and the results obtained with the training of the institute's neurosurgery residents, in order to encourage other teaching centers to adopt similar techniques.

## Materials and methods

**Figure 1:**
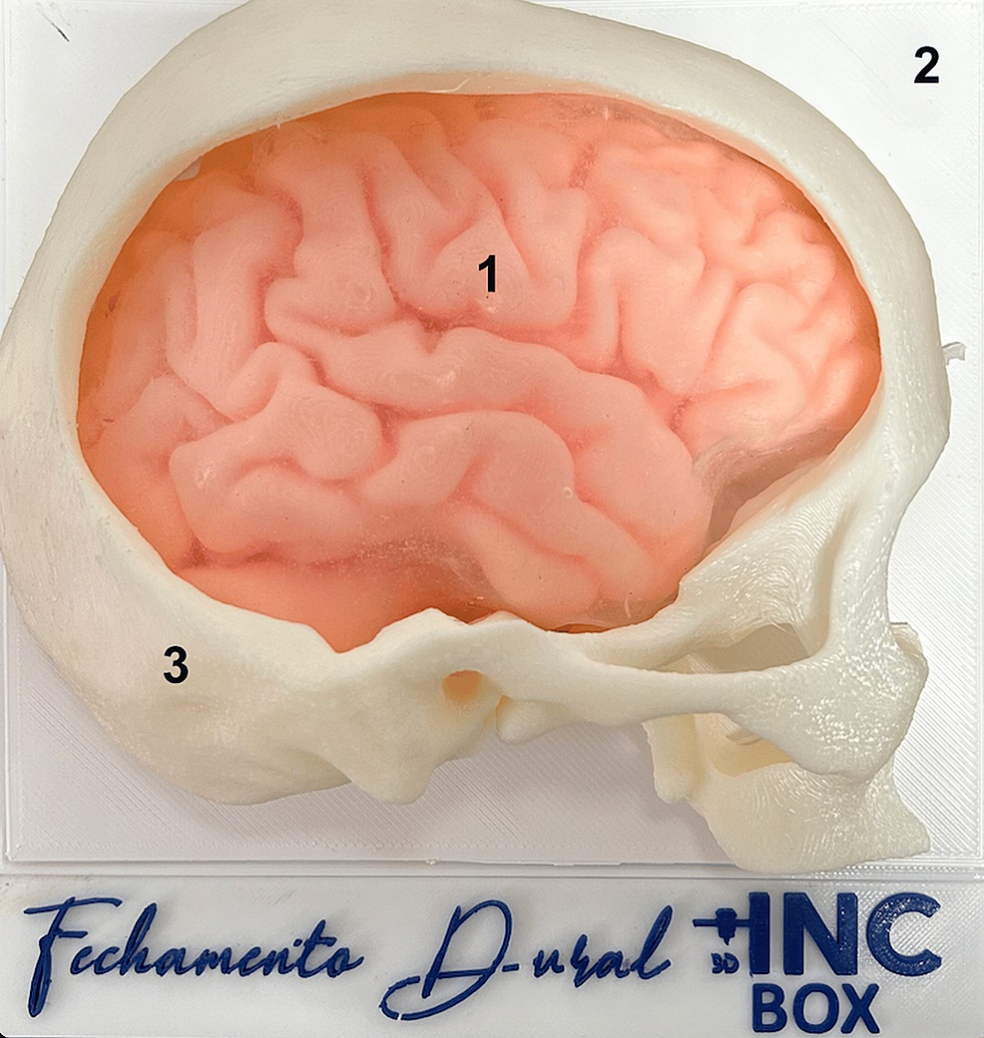
Base of the described simulator base with the brain (1), plate to secure the membrane (2), skull fitting into the base (3)

**Figure 2:**
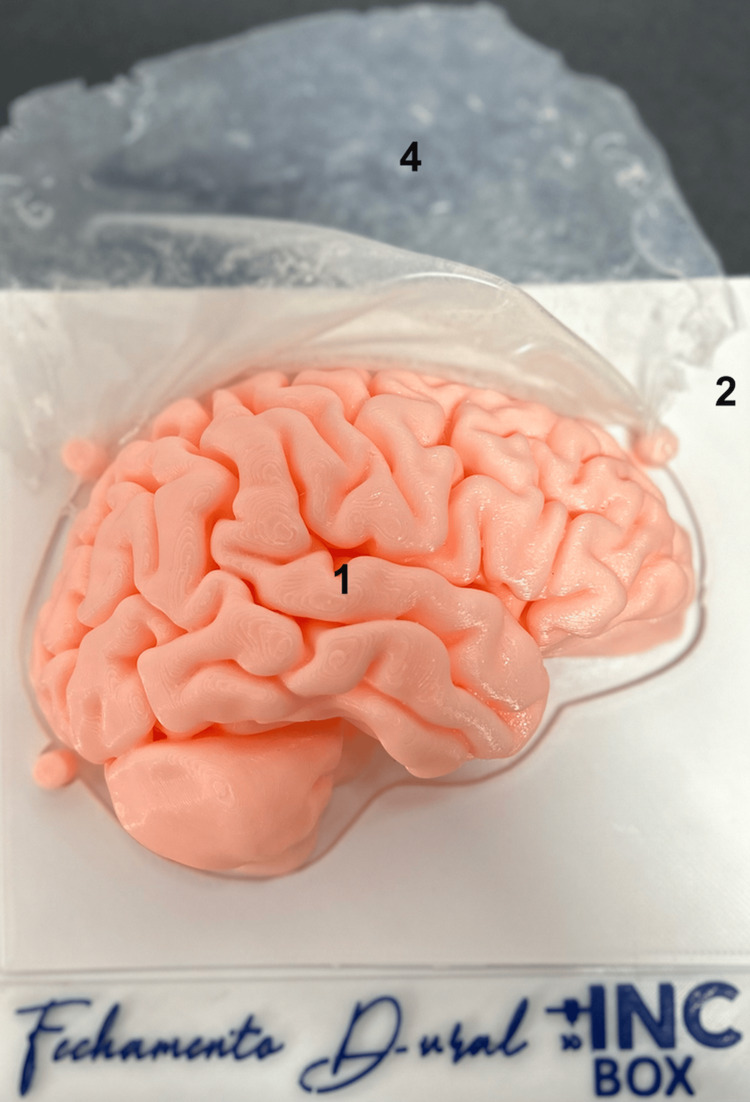
Details of the described simulator base with the brain (1); plate to secure the membrane (2); skull fitting into the base (3); membrane mimicking the dura mater (4)

The authors performed 3D reconstruction of the brain parenchyma and skull, based on magnetic resonance imaging and computed tomography scans, using the 3D Slicer version 5.2 software (developed by the Slicer Community, Cambridge, MA, USA). From the reconstructions, the hemicranium and brain were modeled coupled to a base, allowing the structures to fit together (Figure 1). All modeling was carried out using Autodesk Meshmixer version 3.5 software (developed by Autodesk, Inc., San Rafael, CA, USA). The additive manufacturing of the structures was carried out on a CR5-PRO printer (Creality, Shenzhen, China) using ABS filament (Polymaker, Shanghai, China). The planning of the printing process was carried out using the Ultimaker Cura software version 5.2 (Ultimaker, Utrecht, Netherlands) and the total time for printing the structures was around 16 hours. To simulate the meningeal dura mater, Semi Crystal Platinum Silicone Rubber (Redelease, São Paulo, Brazil) was used, a thin layer was applied to a glass table, and after 24 hours, with complete curing, the membrane was placed between the brain models and skull is used to simulate dural closure (Figure 2).

**Figure 3:**
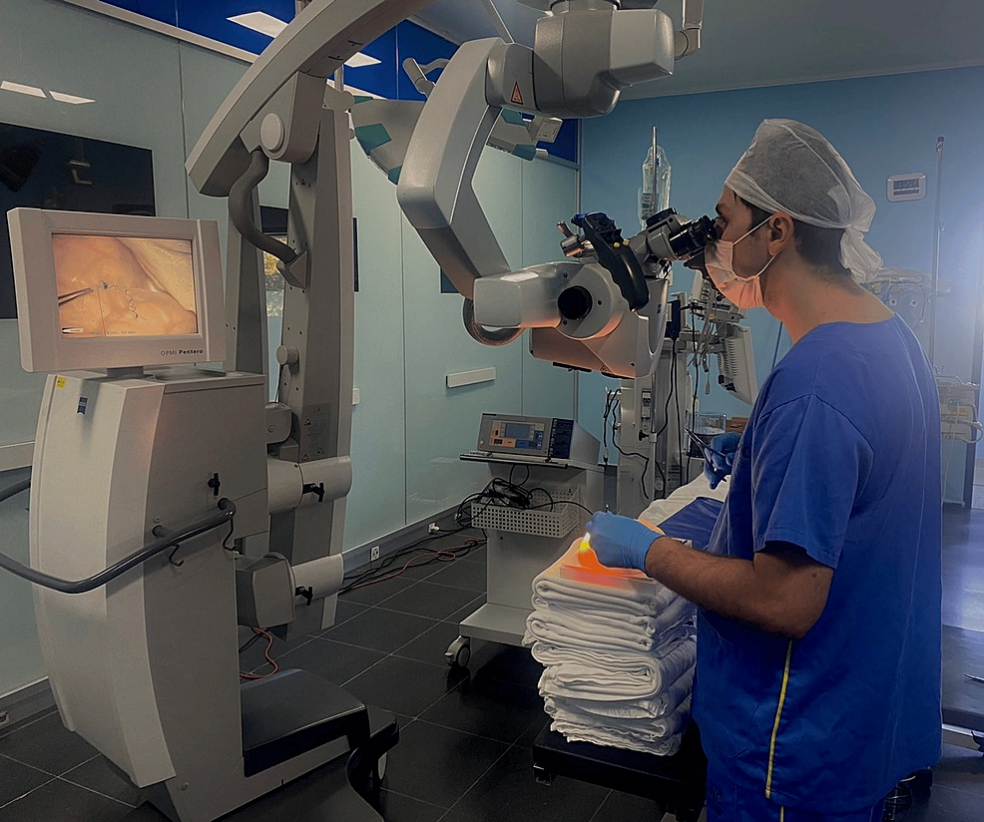
Neurosurgery resident practicing with the described 3D dural closure mold.

After approval by the Research Ethics Committee of Hospital INC (CEPSH-INC), neurosurgery residents from the first to fifth year of residency underwent training using the aforementioned template. The instruction period varied according to the individual needs of the residents, lasting approximately one to two hours, and was conducted in the INC Hospital surgical center facilities, using the surgical microscope (Figure 3). The procedure began with an incision in the silicone rubber that simulated the dura mater, followed by a continuous suture to train dural closure (Video 1). The evaluation of the airtightness of the dural closure was performed through simulated cerebrospinal fluid leakage tests. After the residents performed the sutures, the closure area was subjected to a pressure test using an injected saline solution to check for leaks. The integrity of the suture was visually observed, looking for any fluid escape along the suture line. Additionally, residents were assessed by experienced preceptors who verified the quality and firmness of the sutures performed, providing immediate feedback and scores based on the observed technique and effectiveness.

**Video 1:** Neurosurgery resident practicing with the described 3D dural clousure mold.

The assessment of the knowledge acquired and the effectiveness of the activity was carried out using two virtual forms developed on the Google Forms platform (Table 1), which were filled out by participants before and after carrying out the procedure. Both forms included a section that contained the Free and Informed Consent Form (TCLE), which was duly signed by all participants involved in the research. Both forms employed three assessment methods: a Richter scale from 1 to 10, where 1 represented the minimum and 10 the maximum; binary answer questions ("yes" or "no"); and discursive questions.

**Table 1:** Virtual forms filled out by participants before and after carrying out the procedure

	PRE-ACTIVITY FORM	POST-ACTIVITY FORM
QUESTION		
1	Full Name	Full Name
2	Residency Period	Residency Period
3	How many dural closures have you performed with the assistance and supervision of a neurosurgery preceptor from the beginning of your residency until now?	On a scale of 1 to 10, with 1 being the highest and 10 being the lowest, how would you rate the dural closure training conducted with 3D molds?
4	How many dural closures have you performed from the beginning of your residency until now without the supervision of a neurosurgery preceptor?	On a scale of 1 to 10, with 1 being the highest and 10 being the lowest, how qualified do you consider your technical (theoretical) knowledge to perform this procedure?
5	Do you think more emphasis should be placed on this stage of the surgical procedure?	After the training, on a scale of 1 to 10, with 1 being the highest and 10 being the lowest, how confident do you feel about performing this procedure?
6	If you answered "yes," why? If you answered "no," please leave it blank.	After the training, on a scale of 1 to 10, with 1 being the highest and 10 being the lowest, how insecure do you feel about performing this procedure?
7	On a scale of 1 to 10, with 1 being the highest and 10 being the lowest, how qualified do you consider your technical (theoretical) knowledge to perform this procedure?	On a scale of 1 to 10, with 1 being the highest and 10 being the lowest, how important was the training for your education?
8	On a scale of 1 to 10, with 1 being the highest and 10 being the lowest, how do you evaluate the training on dural closure and suturing in the neurosurgery residency at Hospital INC?	Do you think training with 3D molds is essential for your education?
9	On a scale of 1 to 10, with 1 being the highest and 10 being the lowest, how confident do you feel about performing this procedure?	Would you like more practical training with 3D molds?
10	On a scale of 1 to 10, with 1 being the highest and 10 being the lowest, how insecure do you feel about performing this procedure?	Briefly describe the positive and negative aspects you experienced during the training, both regarding the mold and the activity itself.
11	Do you see a need for more practical training on dural closure?	Any additional considerations? Is there anything you would like to add?

## Results

**Figure 4:**
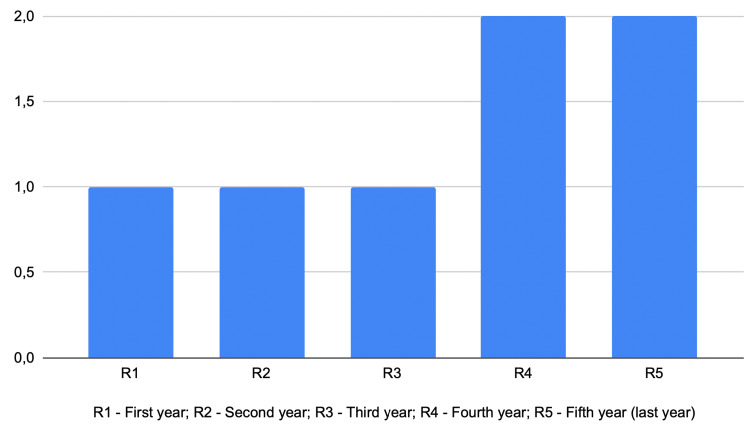
Year of Residency of All The Participants

**Figure 5:**
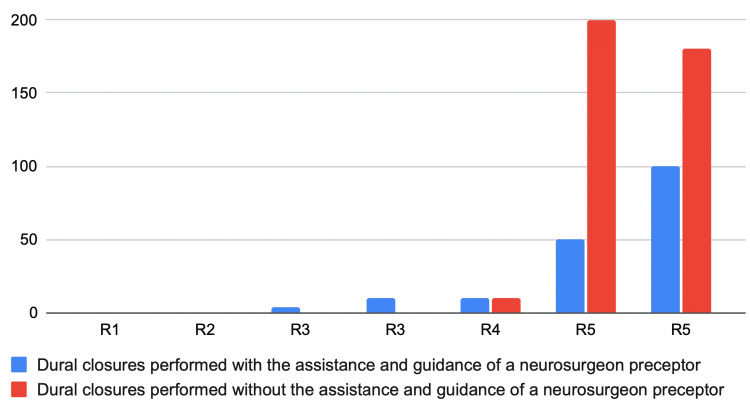
Number of Dural Closures Performed During Residency

A total of seven residents participated in the study, distributed as follows: one from the first year, one from the second year, two from the third year, one from the fourth year and two from the fifth year (Figure 4). The pre-activity form included the assessment of previous contact with the procedure during surgeries, with answers ranging from 0 to 200, depending on the year of residency and the presence or absence of a preceptor surgeon during the procedure (Figure 5). In general, residents in the first two years had little or no direct contact with dural closure, while those in the third and fourth years had some contact only under the supervision of chief neurosurgeons. On the other hand, the fifth- and final-year residents had multiple contacts, acting both as assistants to other neurosurgeons and as independent executors of the procedure.

In the section that used the scale from 1 to 10, it was observed that, after training, for both postgraduate year (PGY)-1 and PGY-2, there was an increase of 2 points in confidence when performing dural closure, and a reduction of 2 points in insecurity during the procedure. PGY-1's theoretical knowledge increased by 2 points, while PGY-2's remained unchanged. For third-year residents, in one case, there was an increase of 2 points in theoretical knowledge and 1 point in confidence when performing the procedure, with insecurity remaining unchanged. In the second case, there was no change in the score. Regarding PGY-4, after the activity, there was an increase of 1 point in theoretical knowledge and a decrease of 2 points in insecurity during dural closure, while confidence in the procedure remained unchanged. Finally, in relation to the two fifth-year residents, in the first case, the values remained unchanged, and in the second case, there was only a 1-point decrease in insecurity when carrying out the procedure (Table 2).

**Table 2:** Comparison between the pre and post-activity forms obtained from the section that used the Richter scale

		PRE-ACTIVITY FORM				
		R1	R2	R3	R4	R5
Question						
7	How qualified do you consider your technical (theoretical) knowledge to perform this procedure?	5	7	7	6	10
				3		8
8	How do you evaluate the training on dural closure and suturing in the neurosurgery residency at Hospital INC?	7	10	9	7	10
				3		6
9	How confident do you feel about performing this procedure?	5	5	7	7	10
				2		8
10	How insecure do you feel about performing this procedure?	5	7	4	4	1
				2		3
		POST-ACTIVITY FORM				
		R1	R2	R3	R4	R5
Question						
3	How would you rate the dural closure training conducted with the 3D model?	10	10	9	8	8
				10		7
4	After the training, how qualified do you consider your technical (theoretical) knowledge to perform this procedure?	7	7	9	7	10
				2		8
5	After the training, how confident do you feel about performing this procedure?	7	7	8	7	10
				2		8
6	After the training, how insecure do you feel about performing this procedure?	5	5	4	3	1
				2		2
7	How important was the training for your neurosurgeon education?	10	10	10	7	10
				1		7
R1 - First-year resident(s) in neurosurgery; R2 - Second-year resident(s) in neurosurgery; R3 - Third-year resident(s) in neurosurgery; R4 - Fourth-year resident(s) in neurosurgery; R5 - Fifth-year resident(s) in neurosurgery						

**Figure 6:**
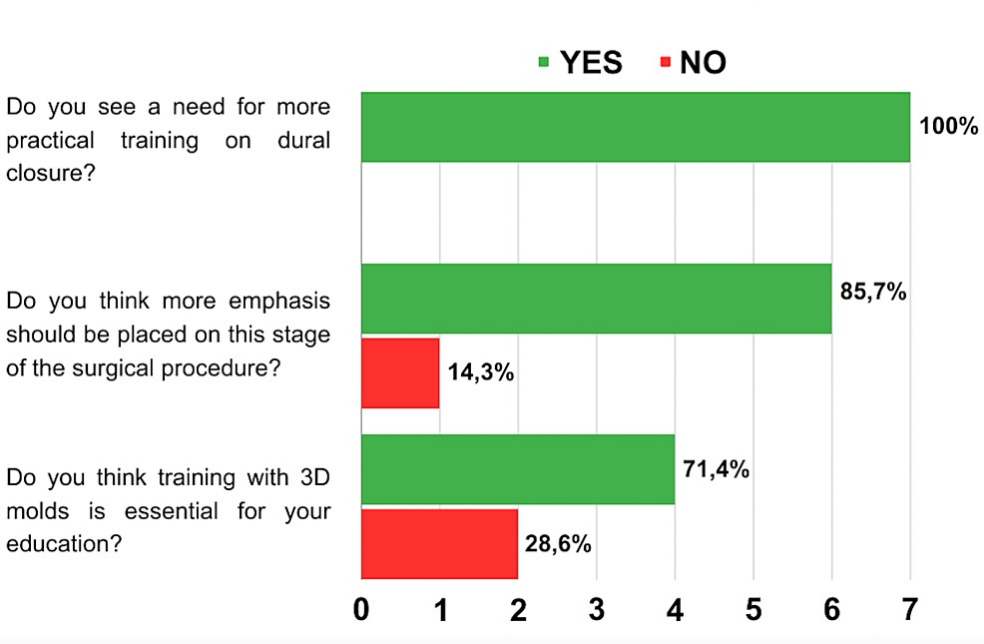
Participants' Responses to The Binary Questions of The Survey

Regarding the binary questions, 100% of participants responded that they would like and see a greater need for more practical training on dural closure. Furthermore, 85.7% believe that there should be greater emphasis on closing the dura mater during surgery, and 71.4% believe that training with 3D molds is essential for their training as a neurosurgeon (Figure 6).

## Discussion

Initially, it is clear that only residents in their final year (fifth year) had successive and autonomous contact with the dural closure procedure, while the others, even with a neurosurgeon preceptor in the field, had restricted contact. These results show that, although medicine and health technologies are advancing rapidly [11-14], new policies defending patients' privacy and autonomy are increasingly limiting the practical activities of resident doctors [11-13].

Furthermore, it was observed that, regardless of the participants' year of residence, everyone showed some level of insecurity when carrying out the procedure, which decreased after carrying out the activity. With the training, there was an improvement in safety when performing the procedure in 71.4% of cases, especially in the surgeons of the first three years of residency.

Similarly, several neurosurgical training bootcamps have been held around the world in recent years, as they provide a low-cost sustainable training model, and have proven to be an effective educational strategy to improve trainees' skills, knowledge and confidence [15,16]. According to studies carried out with 3D simulators, it was discovered that 87.5% of students demonstrated a decrease in dural repair times over consecutive tests, showing more significant progress after fewer attempts, compared to those who received only conventional training [17,18].

Therefrom, not only dexterity and practice were improved, but also the theoretical knowledge of the participants, as two of them reported an improvement in the retention of theoretical information about the technique. Recent meta-analyses have demonstrated that learners who were submitted to a practical approach based on active teaching and execution, also known as the "step-by-step method (SBS)", had a significant improvement in their theoretical background on carrying out and executing steps of the process [19], something that is evident in the study carried out and described in this article.

The requirement to expand applications and activities in simulators becomes evident when all residents participating in the study express the need for more similar meetings. Additionally, 85.7% of participants attributed importance to the study, classifying it as greater than or equal to 7 on the Richter scale (which varies from 1 to 10), in the context of their training in neurosurgery. Among these, four gave the maximum score, while 71.4% considered it essential. In a concurrent investigation, Bakhshi et al. (2022) reported that, among the 22 apprentices evaluated, only one (4.55%) had previously participated in a neurosurgical skills workshop [20]. This scenario reiterates the observation that, although training in 3D simulators is accessible and low-cost, it is a rarity in neurosurgery residency programs, despite representing a response to a need identified by residents around the world.

In this sense, although it is a simple technology, the model developed is innovative, as it allows adequate and repetitive training of direct suturing by neurosurgery residents, addressing the problems of inexperience faced, without violating the ethical principles of patient protection and safety. The process involves performing a direct suture for large or even delicate dural defects [21]. Semi-Crystal Platinum Silicone Rubber (Redelease), used to simulate the dura mater, is a two-component elastomer that vulcanizes at room temperature. This material has a Shore A hardness of 30, an elongation at break of 500%, and a tensile strength of 7.5 MPa, making it an elastic and highly durable product. It is also non-toxic and suitable for creating flexible and robust models. Therefore, its high resistance to wear, tear, and deformation qualifies it for performing dural closure. While there are limitations regarding the exact replication of human nerve structures, the similar composition of this silicone rubber allows for satisfactory learning experiences. Compared to other types of silicone rubber, this material offers superior durability and flexibility, which are essential for realistic surgical simulation [22].

Among the other limitations of the study are: (1) the participation restricted to neurosurgical residents from a single hospital institution, (2) the lack of a control group for a longitudinal analysis of training developments, (3) the adoption of a single mold model, and (4) the restriction imposed by the small sample size.

Future perspectives

The progressive development of bioprinters makes it possible to generate organic materials using polymers that are compatible with the human body and biodegradable, such as collagen and hyaluronic acid. These substances, when mixed in an aqueous solution, transform into a hydrogel, and, after the addition of human cells, biotin is formed, which is a precursor to biological tissues. In other words, the use of biopolymers, hydrogels and nano-structural properties of human tissues allows the extracellular environment and microenvironment of the printed tissue to resemble real tissues [23]. In addition to expanding the ability to personalize treatment using the most anatomically appropriate structure and increasing patient compliance, this technology, by having an extremely high similarity index to human tissues, allows for an increasingly realistic simulation of learning, through the creation of of sophisticated prototypes that, in the future, will be indistinguishable from real fabrics [24]. However, the low bioprinting rate of complex structures, especially in multimaterial biofabrication, and the distance between cells and capillaries still represent a challenge, requiring further studies for resolving solutions [25-27].

## Conclusions

Adequate training for dural closure is essential to avoid serious complications in neurosurgery. Surgical simulation, such as the use of 3D models, has been shown to be an effective educational strategy to improve the skills of neurosurgeons in training and prevent the progression of adverse events. Although the use of simulation materials similar to human tissues, such as silicone rubber, provides satisfactory learning, there are still limitations regarding their similarity to human tissues. However, with the advent of bioprinting, such difficulties can be overcome, and the trend of using prototypes in medical education is in an inevitable process of rise.
